# *N*-(2-Chloro­phen­yl)-*N*′-(2-methyl­phen­yl)succinamide

**DOI:** 10.1107/S1600536811035616

**Published:** 2011-09-14

**Authors:** B. S. Saraswathi, Sabine Foro, B. Thimme Gowda

**Affiliations:** a Department of Chemistry, Mangalore University, Mangalagangotri 574 199, Mangalore, India; b Institute of Materials Science, Darmstadt University of Technology, Petersenstrasse 23, D-64287 Darmstadt, Germany

## Abstract

In the title compound, C_17_H_17_ClN_2_O_2_, the asymmetric unit contains half a mol­ecule with a centre of symmetry at the mid-point of the central C—C bond. The conformations of the amide O atoms are *anti* to the methyl­ene atoms. Further, the N—H bonds in the amide fragments are *anti* to the *ortho*-chloro/methyl groups in the adjacent benzene rings. The dihedral angle between the benzene ring and the NH—C(O)—CH_2_ segment in the two halves of the mol­ecule is 62.0 (2)°. In the crystal, a series of N—H⋯O inter­molecular hydrogen bonds link the mol­ecules into column-like infinite chains along the *a* axis. The methyl and Cl groups are disordered with respect to the *ortho* positions of the benzene ring, with site-occupation factors of 0.5 each.

## Related literature

For our studies on the effects of substituents on the structures of *N*-(ar­yl)-amides, see: Bhat & Gowda (2000 ▶); Gowda *et al.* (2007*a* ▶); Saraswathi *et al.* (2011**a* ▶,*b* ▶,c* ▶); and on the structures of *N*-(ar­yl)-methane­sulfonamides, see: Gowda *et al.* (2007*b* ▶). For similar structures, see: Pierrot *et al.* (1984 ▶). For restrained geometry, see: Nardelli (1999 ▶).
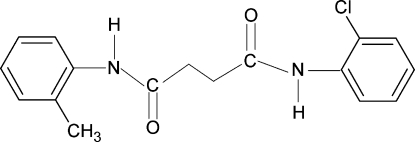


## Experimental

### 

#### Crystal data

C_17_H_17_ClN_2_O_2_
*M* *_r_* = 316.78
Monoclinic, 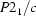

*a* = 11.541 (3) Å
*b* = 7.908 (2) Å
*c* = 8.798 (2) Å
β = 102.55 (2)°
*V* = 783.8 (3) Å^3^
*Z* = 2
Mo *K*α radiation
μ = 0.25 mm^−1^
*T* = 293 K
0.48 × 0.04 × 0.04 mm

#### Data collection

Oxford Diffraction Xcalibur diffractometer with a Sapphire CCD detector
Absorption correction: multi-scan (*CrysAlis RED*; Oxford Diffraction, 2009 ▶) *T*_min_ = 0.889, *T*_max_ = 0.990
2598 measured reflections
1391 independent reflections
824 reflections with *I* > 2σ(*I*)
*R*_int_ = 0.044

#### Refinement

*R*[*F* ^2^ > 2σ(*F* ^2^)] = 0.105
*wR*(*F* ^2^) = 0.164
*S* = 1.28
1391 reflections
112 parameters
9 restraints
H atoms treated by a mixture of independent and constrained refinement
Δρ_max_ = 0.26 e Å^−3^
Δρ_min_ = −0.31 e Å^−3^

### 

Data collection: *CrysAlis CCD* (Oxford Diffraction, 2009 ▶); cell refinement: *CrysAlis RED* (Oxford Diffraction, 2009 ▶); data reduction: *CrysAlis RED*; program(s) used to solve structure: *SHELXS97* (Sheldrick, 2008 ▶); program(s) used to refine structure: *SHELXL97* (Sheldrick, 2008 ▶); molecular graphics: *PLATON* (Spek, 2009 ▶); software used to prepare material for publication: *SHELXL97*.

## Supplementary Material

Crystal structure: contains datablock(s) I, global. DOI: 10.1107/S1600536811035616/vm2112sup1.cif

Structure factors: contains datablock(s) I. DOI: 10.1107/S1600536811035616/vm2112Isup2.hkl

Supplementary material file. DOI: 10.1107/S1600536811035616/vm2112Isup3.cml

Additional supplementary materials:  crystallographic information; 3D view; checkCIF report

## Figures and Tables

**Table 1:** Hydrogen-bond geometry (Å, °)

*D*—H⋯*A*	*D*—H	H⋯*A*	*D*⋯*A*	*D*—H⋯*A*
N1—H1*N*⋯O1^i^	0.85 (2)	2.00 (2)	2.846 (5)	170 (5)

Symmetry code: (i) 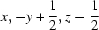.

**Fig. 1.**
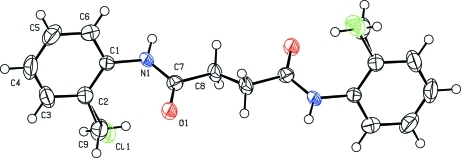
Molecular structure of the title compound, showing the atom labelling scheme and displacement ellipsoids are drawn at the 50% probability level. Symmetry operation to generate second half: -x + 1, -y, -z.

**Fig. 2.**
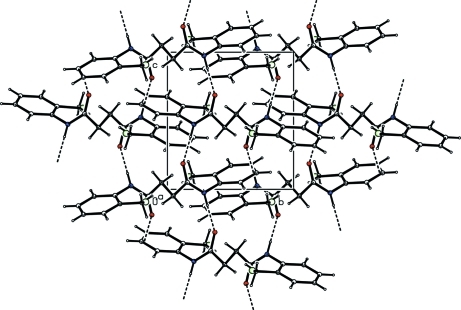
Molecular packing of the title compound with hydrogen bonding shown as dashed lines.

## supplementary crystallographic information

### Comment

The amide and sulfonamide moieties are important constituents of many biologically significant compounds. As part of our studies on the substituent effects on the structures of this class of compounds (Bhat & Gowda, 2000; Gowda *et al.*, 2007**a*,b*; Saraswathi *et al.*, 2011**a*,*b*,c*), in the present work, the structure of *N*-(2-Chlorophenyl),*N*-(2-methylphenyl)-succinamide (I) has been determined (Fig.1). The asymmetric unit of (I) contains half a molecule with a center of symmetry at the mid-point of the central C—C bond, similar to that obseved in bis(2-chlorophenylaminocarbonylmethyl)disulfide (II) (Pierrot *et al.*, 1984), *N*-(3-Chlorophenyl),*N*-(3-methylphenyl)- succinamide (III) (Saraswathi *et al.*, 2011*c*), *N*,*N*-bis(2-chlorophenyl)-succinamide (IV) (Saraswathi *et al.*, 2011*b*) and *N*,*N*-bis(2-methylphenyl)-succinamide dihydrate (V) (Saraswathi *et al.*, 2011*a*).

The conformations of the amide O atoms are *anti* to the H atoms attached to the adjacent C atoms. Further, the conformations of the N—H bonds in the amide fragments are *anti* to the *ortho*- chloro/methyl groups in the adjacent benzene rings, similar to that observed with respect to the *meta*-chloro/methyl groups in (III), and the *anti* conformations observed with respect to the *ortho*-methyl groups in (V), but contrary to the *syn* conformations observed between the N—H bonds in the amide fragments and the *ortho*-chloro groups in the adjacent benzene rings of (IV).

Further, C1—N1—C7—C8 and C1a—N1a—C7a—C8a segments in (I) are nearly linear, similar to those observed in (III), (IV) and (V). The torsion angles of C2—C1—N1—C7 and C6—C1—N1—C7 are -64.3 (8)° and 115.8 (6)°, compared to the values of -43.2 (4)° and 138.6 (3)° in (III), -47.6 (6)° and 133.7 (4)° in (IV) and -64.0 (4)° and 117.6 (3)° in (V).

The dihedral angle between the benzene ring and the NH—C(O)—CH_2_ segment in the two halves of the molecule is 62.0 (2)°, compared to the values of 43.5 (1)° in (III), 47.0 (2)° in (IV) and 62.1 (2)° in (V).

The packing of molecules in the crystal linked by of N—H···O hydrogen bonds (Table 1) is shown in Fig. 2.

### Experimental

Succinic anhydride (0.01 mol) in toluene (25 ml) was treated drop wise with 2-chloroaniline (0.01 mol) also in toluene (20 ml) with constant stirring. The resulting mixture was stirred for one hour and set aside for an additional hour at room temperature for completion of the reaction. The mixture was then treated with dilute hydrochloric acid to remove unreacted 2-chloroaniline. The resultant solid *N*-(2-chlorophenyl)-succinamic acid was filtered under suction and washed thoroughly with water to remove the unreacted succinic anhydride and succinic acid. The compound was recrystallized to constant melting point from ethanol. The purity of the compound was checked by elemental analysis and characterized by its infrared and NMR spectra.

The *N*-(2-chlorophenyl)succinamic acid obtained was then treated with phosphorous oxychloride and excess of 2-methylaniline at room temperature with constant stirring. The resultant mixture was stirred for 4 h, kept aside for additional 6 h for completion of the reaction and poured slowly into crushed ice with constant stirring. It was kept aside for a day. The resultant solid, *N*-(2-chlorophenyl), *N*-(2-methylphenyl)-succinamide was filtered under suction, washed thoroughly with water, dilute sodium hydroxide solution and finally with water. It was recrystallized to constant melting point from a mixture of acetone and toluene (3:1 *v*/*v*). The compound was characterized by its infrared and NMR spectra.

Needle like colorless single crystals used in X-ray diffraction studies were grown in a mixture of acetone and toluene (3:1 *v*/*v*) at room temperature.

### Refinement

The H atom of the NH group was located in a difference map and later restrained to the distance N—H = 0.86 (2) Å. The other H atoms were positioned with idealized geometry using a riding model with the aromatic C—H = 0.93 Å, methyl C—H = 0.97 Å, and the methylene C—H = 0.97 Å.

All H atoms were refined with isotropic displacement parameters. The *U*_iso_(H) values were set at 1.2*U*_eq_(C-aromatic, N) and 1.5*U*_eq_(*C*-methyl).

The residual electron-density features are located in the region of Cl1 and C2. The highest peak is 0.66 Å from Cl1 and the deepest hole is 0.23 Å from C2.

C9 and Cl1 are disordered and were refined using a split model. The corresponding site-occupation factors were fixed to 0.50:0.50. The U^ij^ components of C9 were restrained to approximate isotropic behavoir (Nardelli, 1999). The bond lengths C2–C9 and C2–Cl1 were restrained to 1.54 (1) and 1.74 (1) Å, respectively.

### Crystal data

**Table:**

C_17_H_17_ClN_2_O_2_	*F*(000) = 332
*M**_r_* = 316.78	*D*_x_ = 1.342 Mg m^−^^3^
Monoclinic, *P*2_1_/*c*	Mo *K*α radiation, λ = 0.71073 Å
Hall symbol: -P 2ybc	Cell parameters from 611 reflections
*a* = 11.541 (3) Å	θ = 2.6–27.9°
*b* = 7.908 (2) Å	µ = 0.25 mm^−^^1^
*c* = 8.798 (2) Å	*T* = 293 K
β = 102.55 (2)°	Needle, colourless
*V* = 783.8 (3) Å^3^	0.48 × 0.04 × 0.04 mm
*Z* = 2	

### Data collection

**Table:**

Oxford Diffraction Xcalibur diffractometer with a Sapphire CCD detector	1391 independent reflections
Radiation source: fine-focus sealed tube	824 reflections with *I* > 2σ(*I*)
graphite	*R*_int_ = 0.044
Rotation method data acquisition using ω scans	θ_max_ = 25.2°, θ_min_ = 3.2°
Absorption correction: multi-scan (*CrysAlis RED*; Oxford Diffraction, 2009)	*h* = −9→13
*T*_min_ = 0.889, *T*_max_ = 0.990	*k* = −9→5
2598 measured reflections	*l* = −9→10

### Refinement

**Table:**

Refinement on *F*^2^	Primary atom site location: structure-invariant direct methods
Least-squares matrix: full	Secondary atom site location: difference Fourier map
*R*[*F*^2^ > 2σ(*F*^2^)] = 0.105	Hydrogen site location: inferred from neighbouring sites
*wR*(*F*^2^) = 0.164	H atoms treated by a mixture of independent and constrained refinement
*S* = 1.28	*w* = 1/[σ^2^(*F*_o_^2^) + (0.0062*P*)^2^ + 2.1127*P*] where *P* = (*F*_o_^2^ + 2*F*_c_^2^)/3
1391 reflections	(Δ/σ)_max_ < 0.001
112 parameters	Δρ_max_ = 0.26 e Å^−^^3^
9 restraints	Δρ_min_ = −0.31 e Å^−^^3^

### Special details

**Table:**

Geometry. All e.s.d.'s (except the e.s.d. in the dihedral angle between two l.s. planes) are estimated using the full covariance matrix. The cell e.s.d.'s are taken into account individually in the estimation of e.s.d.'s in distances, angles and torsion angles; correlations between e.s.d.'s in cell parameters are only used when they are defined by crystal symmetry. An approximate (isotropic) treatment of cell e.s.d.'s is used for estimating e.s.d.'s involving l.s. planes.
Refinement. Refinement of *F*^2^ against ALL reflections. The weighted *R*-factor *wR* and goodness of fit *S* are based on *F*^2^, conventional *R*-factors *R* are based on *F*, with *F* set to zero for negative *F*^2^. The threshold expression of *F*^2^ > σ(*F*^2^) is used only for calculating *R*-factors(gt) *etc*. and is not relevant to the choice of reflections for refinement. *R*-factors based on *F*^2^ are statistically about twice as large as those based on *F*, and *R*- factors based on ALL data will be even larger.

### Fractional atomic coordinates and isotropic or equivalent isotropic displacement parameters (Å^2^)

**Table:**

	*x*	*y*	*z*	*U*_iso_*/*U*_eq_	Occ. (<1)
C1	0.2685 (5)	0.4153 (7)	0.0447 (6)	0.0318 (14)	
C2	0.1709 (5)	0.3763 (7)	0.1055 (6)	0.0370 (15)	
C3	0.1179 (6)	0.5036 (9)	0.1745 (7)	0.0526 (18)	
H3	0.0527	0.4781	0.2165	0.063*	
C4	0.1595 (6)	0.6660 (9)	0.1821 (8)	0.0579 (19)	
H4	0.1227	0.7495	0.2293	0.069*	
C5	0.2556 (6)	0.7058 (8)	0.1202 (8)	0.0557 (19)	
H5	0.2839	0.8163	0.1248	0.067*	
C6	0.3099 (6)	0.5805 (8)	0.0512 (7)	0.0489 (18)	
H6	0.3747	0.6071	0.0087	0.059*	
C7	0.3869 (5)	0.1569 (7)	0.0508 (6)	0.0303 (13)	
C8	0.4477 (5)	0.0460 (7)	−0.0481 (6)	0.0353 (14)	
H8A	0.3913	−0.0357	−0.1034	0.042*	
H8B	0.4743	0.1154	−0.1249	0.042*	
C9	0.110 (3)	0.203 (3)	0.095 (5)	0.057 (12)	0.50
H9A	0.1531	0.1238	0.0459	0.068*	0.50
H9B	0.0301	0.2127	0.0350	0.068*	0.50
H9C	0.1083	0.1639	0.1980	0.068*	0.50
N1	0.3288 (4)	0.2897 (6)	−0.0256 (5)	0.0346 (12)	
H1N	0.338 (4)	0.316 (7)	−0.116 (3)	0.041*	
O1	0.3888 (3)	0.1237 (5)	0.1871 (4)	0.0410 (11)	
Cl1	0.1161 (9)	0.1725 (10)	0.0927 (15)	0.057 (2)	0.50

### Atomic displacement parameters (Å^2^)

**Table:**

	*U*^11^	*U*^22^	*U*^33^	*U*^12^	*U*^13^	*U*^23^
C1	0.038 (3)	0.033 (3)	0.026 (3)	0.007 (3)	0.011 (3)	0.005 (3)
C2	0.039 (4)	0.037 (4)	0.037 (4)	0.006 (3)	0.012 (3)	0.002 (3)
C3	0.052 (4)	0.062 (5)	0.050 (4)	0.021 (4)	0.025 (3)	0.002 (4)
C4	0.070 (5)	0.046 (5)	0.061 (5)	0.027 (4)	0.021 (4)	−0.005 (4)
C5	0.075 (5)	0.028 (4)	0.065 (5)	0.009 (4)	0.018 (4)	0.003 (3)
C6	0.063 (5)	0.038 (4)	0.052 (4)	0.003 (3)	0.027 (4)	0.011 (3)
C7	0.039 (3)	0.029 (3)	0.024 (3)	0.000 (3)	0.009 (3)	−0.002 (3)
C8	0.044 (4)	0.039 (3)	0.026 (3)	0.010 (3)	0.013 (3)	−0.006 (3)
C9	0.063 (15)	0.051 (13)	0.057 (14)	0.014 (8)	0.016 (9)	−0.003 (8)
N1	0.048 (3)	0.037 (3)	0.025 (3)	0.010 (2)	0.023 (2)	0.004 (2)
O1	0.065 (3)	0.041 (2)	0.023 (2)	0.017 (2)	0.021 (2)	0.0045 (19)
Cl1	0.046 (3)	0.051 (3)	0.084 (5)	−0.010 (3)	0.031 (3)	−0.008 (3)

### Geometric parameters (Å, °)

**Table:**

C1—C2	1.383 (7)	C6—H6	0.9300
C1—C6	1.388 (7)	C7—O1	1.223 (6)
C1—N1	1.429 (6)	C7—N1	1.345 (7)
C2—C3	1.384 (7)	C7—C8	1.512 (7)
C2—C9	1.535 (10)	C8—C8^i^	1.503 (10)
C2—Cl1	1.725 (7)	C8—H8A	0.9700
C3—C4	1.367 (9)	C8—H8B	0.9700
C3—H3	0.9300	C9—H9A	0.9600
C4—C5	1.375 (8)	C9—H9B	0.9600
C4—H4	0.9300	C9—H9C	0.9600
C5—C6	1.382 (8)	N1—H1N	0.853 (19)
C5—H5	0.9300		
			
C2—C1—C6	119.7 (5)	C1—C6—H6	119.7
C2—C1—N1	121.8 (5)	O1—C7—N1	124.0 (5)
C6—C1—N1	118.5 (5)	O1—C7—C8	121.9 (5)
C1—C2—C3	118.8 (5)	N1—C7—C8	114.1 (4)
C1—C2—C9	125.3 (16)	C8^i^—C8—C7	111.9 (5)
C3—C2—C9	115.8 (15)	C8^i^—C8—H8A	109.2
C1—C2—Cl1	120.0 (5)	C7—C8—H8A	109.2
C3—C2—Cl1	121.2 (6)	C8^i^—C8—H8B	109.2
C9—C2—Cl1	5.9 (18)	C7—C8—H8B	109.2
C4—C3—C2	121.4 (6)	H8A—C8—H8B	107.9
C4—C3—H3	119.3	C2—C9—H9A	109.5
C2—C3—H3	119.3	C2—C9—H9B	109.5
C3—C4—C5	120.0 (6)	H9A—C9—H9B	109.5
C3—C4—H4	120.0	C2—C9—H9C	109.5
C5—C4—H4	120.0	H9A—C9—H9C	109.5
C4—C5—C6	119.5 (6)	H9B—C9—H9C	109.5
C4—C5—H5	120.3	C7—N1—C1	124.3 (4)
C6—C5—H5	120.3	C7—N1—H1N	121 (4)
C5—C6—C1	120.5 (5)	C1—N1—H1N	114 (4)
C5—C6—H6	119.7		
			
C6—C1—C2—C3	−1.2 (9)	C3—C4—C5—C6	−0.3 (11)
N1—C1—C2—C3	178.9 (5)	C4—C5—C6—C1	−0.3 (10)
C6—C1—C2—C9	175 (2)	C2—C1—C6—C5	1.1 (9)
N1—C1—C2—C9	−5(2)	N1—C1—C6—C5	−179.0 (6)
C6—C1—C2—Cl1	178.0 (7)	O1—C7—C8—C8^i^	−28.1 (9)
N1—C1—C2—Cl1	−1.9 (9)	N1—C7—C8—C8^i^	153.7 (6)
C1—C2—C3—C4	0.6 (10)	O1—C7—N1—C1	4.3 (9)
C9—C2—C3—C4	−176 (2)	C8—C7—N1—C1	−177.6 (5)
Cl1—C2—C3—C4	−178.7 (7)	C2—C1—N1—C7	−64.2 (8)
C2—C3—C4—C5	0.2 (11)	C6—C1—N1—C7	115.8 (6)

Symmetry codes: (i) −*x*+1, −*y*, −*z*.

### Hydrogen-bond geometry (Å, °)

**Table:**

*D*—H···*A*	*D*—H	H···*A*	*D*···*A*	*D*—H···*A*
N1—H1N···O1^ii^	0.85 (2)	2.00 (2)	2.846 (5)	170 (5)

Symmetry codes: (ii) *x*, −*y*+1/2, *z*−1/2.
